# Heart transplantation in atrial switch and in congenitally corrected transposition of the great arteries

**DOI:** 10.1186/1749-8090-8-S1-O305

**Published:** 2013-09-11

**Authors:** E Delmo Walter, R Hetzer

**Affiliations:** 1 Cardiothoracic Surgery, Deutsches Herzzentrum Berlin, Berlin, Germany

## Background

Patients with congenitally corrected transposition of the great arteries (ccTGA) and those who underwent atrial switch eventually face systemic ventricular failure due to deterioration of the morphological and structural function. Heart transplantation is at present one of the two best available options. We report our institutional experience of heart transplantation in this group, with emphasis on surgical techniques, risk factors and long-term outcome.

## Methods

Between 1992 and 2011, 21 patients (mean age: 21.68±6, range 1.4-27 years) with TGA (L-TGA=7; D-TGA=8; ccTGA=6) underwent heart transplantation for end-stage heart failure. Previous operations were Senning procedure (n=8), Mustard operation (n=5), double switch (n=2), arterial switch (n=2), Fontan operation (n=1), mitral and tricuspid valve replacement (n=1) and palliative shunting (n=2). Six patients had pulmonary hypertension.

## Results

Previous surgeries, alterations in the atrial anatomy, other anatomic abnormalities and the presence of intra-atrial conduits required technical modifications in harvesting and implantation. Postoperative morbidity was hemorrhage which was easily controlled. Cause of early mortality was severe pulmonary hypertension. Late mortality was due to graft dysfunction. At a mean follow-up of 5.9 years (1-22.8 years), overall survival rates are 78.6%, 57.1% and 42.8%, at 1, 5 and 20 years.

## Conclusions

Previous operations did not produce major technical problems in heart transplantation. Pulmonary hypertension is the only risk factor for mortality. The long-term survival rate is highly satisfactory.

